# Mapping the core competencies and entrustable professional activities of medical ethics for faculty members

**DOI:** 10.1186/s12909-023-04305-1

**Published:** 2023-06-05

**Authors:** Jannat Mashayekhi, Mahboobeh Khabaz Mafinejad

**Affiliations:** 1 Medical Ethics Department, Medical school, Quran and Hadith Research Center, Baqiyatallah University of Medical Sciences, Tehran, Iran; 2 Health Professions Education Research Center, Education Development Center, Department of Medical Education, Tehran University of Medical Sciences, Tehran, Iran

**Keywords:** Medical Ethics, Entrustable Professional Activities, Competencies, Faculty Members

## Abstract

**Introduction:**

The present study aimed to develop core competencies and Entrustable Professional Activities (EPAs) for faculty members through participating in faculty development programs in medical ethics.

**Methods:**

This study included five stages. First, categories and subcategories were extracted based on the literature review and interviews with 14 experts and through inductive content analysis. Second, the content validity of the core competency list was checked by 16 experts using qualitative and quantitative approaches. Third, based on the previous phase, a framework for EPAs was developed by the taskforce in two sessions through consensus. Fourth, the content validity of the list of EPAs was compiled based on a three-point Likert 11 medical ethics experts from necessity and relevance perspectives. Fifth, EPAs were mapped by ten experts to the developed core competencies.

**Results:**

After conducting the literature review and interviews, 295 codes were extracted, which were further classified into six categories and 18 subcategories. Finally, five core competencies and 23 EPAs were developed. The core competencies include “Teaching medical ethics”, “Research and scholarship in the field of medical ethics”, “Communication skills”, “Moral reasoning”, and “Policy-making, decision-making, and ethical leadership”.

**Conclusion:**

Medical teachers can be effective in the moralizing healthcare system. Findings showed that faculty members should acquire core competencies and EPAs for proficiently integrating medical ethics into curricula. Faculty development programs can be designed in medical ethics for faculty members to help them to acquire core competencies and EPAs.

## Introduction

Medical ethics is a core competency in medical education [1], and faculty members can be significantly effective in conveying moral values ​​to students [2]. Faculty members tackle medical ethics challenges in clinical service delivery, education, and research [3]. Thus, successfully providing clinical services to patients depends not only on medical knowledge and skills but also on the appropriate and ethical relationship with patients, respect for patients, confidentiality and privacy, honesty with patients, as well as maintaining patients’ trust [4], that also lead to reducing medical errors [5]. Moreover, the majority of medical knowledge comes from scientific research, and hence, appropriate teaching of research ethics is a duty of faculty members in addition to presenting knowledge regarding the code of conduct and guidelines in healthcare practice [6, 7]. Moreover, observance of ethical principles in providing clinical services and research by faculty members, as role models, affects students as it informally conveys their moral values through hidden curriculum [8, 9]. Observing professional behaviors through such role modeling, which can later have positive effects on improving patient care delivery [10], is often considered stronger than direct teaching in formal curricula [11]. Through role modeling, medical teachers affect medical students’ attitudes, norms, and beliefs, as well as help them develop professional values [12]. However, direct teaching of medical ethics to students is required [13], and without it, students may not achieve moral growth and decision-making competency to resolve ethical dilemmas [14].

Faculty members might consider themselves incapable of teaching and conveying moral values ​​and professional behaviors to students because they have not received formal education in medical ethics [15]. Eckles et al. showed that faculty members could not play their role in improving medical ethics competency of students, and it needs medical faculty members should learn about their role in the field of medical ethics through participation in relevant faculty development programs [16]. Evidence shows that medical ethics education for them has been neglected [17] or inadequate [18]. Hence, to design effective medical ethics programs for faculty members, core competencies in medical ethics should be highlighted, since, currently, competency-based education is considered a fundamental strategy for training and assessment [19]. The competencies are the quality of people’s ability based on their knowledge, attitude, and skills, but Entrustable Professional Activities (EPAs) are units of professional practice and require the integration of competencies to perform professional tasks. Possessing core competencies and being qualified for EPAs is the aim of medical education [20].

Literature on the development of competencies and EPAs of medical ethics for faculty members has so far been very limited, and no definition was uniformly agreed upon [21]. Furthermore, faculty members need to adapt themselves to have related, required, and expected competencies in various training situations. This study aimed to define core competencies and Entrustable Professional Activities (EPAs) for faculty members that need to be accommodated in the faculty development programs in the field of medical ethics.

## Methods and materials

This study was conducted in five stages at Tehran University of Medical Sciences (TUMS) in Iran in 2018–2020.

### A - Stage 1: Literature review

**A1**: Narrative review of the literature was conducted using the following keywords: “fellowship”, “faculty development”, “curriculum”, “medical ethics”, and “bioethics” in PubMed, Web of Science, and Scopus databases on English articles. By critical review of the titles and abstracts of articles, those related to undergraduate medical education and other health professional training were excluded. However, articles about faculty members were included, and abstracts presented at conferences or other meetings were disregarded. Then, the remaining articles were thematically reviewed and analyzed.

#### A2

A rapid review of gray literature was done on Google to identify medical ethics fellowship and faculty development programs, and the data unrelated to medical ethics faculty development programs or lacking access to details of programs were excluded. The data, then, were analyzed using a directed content analysis approach [22].

### A3: Conducting interviews

A purposive sampling and semi-structured individual interviews were conducted to achieve medical ethics tasks and core competencies expected from faculty members. Fourteen participants were selected purposively from different disciplines including clinicians, medical ethicists, and managers. The experts had related experiences or educational activities in medical ethics for at least five years. Two questions were prepared before conducting the interviews and probing questions were asked based on the interviewee’s answers if needed. In this phase, we have sought the view of experts about the main questions including:

1. What core competencies are needed for a faculty member in the field of medical ethics?
2. What are the expected tasks of a faculty member in the field of medical ethics?

The time and place were determined based on the participant’s preference. Semi-structured interviews lasted from 30 to 90 minutes depending on interactions between the interviewer and interviewee. The content of the interviews was recorded one by one. After conducting the interview, the texts were transcribed. Data collection and analysis were concurrent. Data collection was continued till data saturation (no new data emerged). In this phase, inductive content analysis was applied [23–25].

### B- Stage 2: Holding the expert panel

A list of competencies was compiled to be assessed by the expert panel. Participants from different disciplines were purposively selected with maximum variation in the specialty (medical ethicist, clinical specialists and managers) and gender. The 16 recruited experts who had experience in the field of medical ethics attended a session that lasted about two and a half hours. At the beginning of the session, a moderator explained session goals and presented a list of expected competencies derived from the previous phases. Then, participants were asked to comment further on them, and the session continued to reach a consensus on core competencies by calculating quantitative content validity indicators (CVR, CVI). CVR was calculated using the Lawshe method and CVI using the Waltz & Bausell method, respectively, and CVR and CVI values ranged from + 1 to -1. Competencies with an I-CVI of 0.79 or higher remained in the final list, and competencies with an I-CVR of 0.49 or higher were considered evidence of sufficient content validity.

### C- Stage 3: Developing EPAs framework

In this stage, a taskforce, composed of experts from medical education and medical ethics, was established to develop a set of EPAs. Core EPAs framework drafted by taskforce based on the extracted codes from prior literature review and interviews using conventional content analysis. The codes of the interviews and literature review were assessed by two researchers in two 2-hour sessions together. Whenever there were disagreements in codes, these were reconciled collaboratively and discussed until a consensus was reached.

### D- Stage 4: Validating EPAs framework

This stage assessed the validity of EPAs. To evaluate EPAs, experts examined EPAs from necessity and relevancy. The list of EPAs was sent to 11 experts in medical ethics through an e-mail to respond to the questionnaire based on a three-point Likert scale (highly important, moderately important and less important). Finally, items with scores of 70% or higher remained on the final list.

### E- Stage 5: Mapping EPAs to core competencies

Mapping EPAs to core competencies is intended to create consistency in education and assessment of EPAs along with the core competencies and to address the shortcomings of a snapshot, subjective judgments of complex tasks. Experts consisting of 10 faculty members of medical ethics and medical education mapped EPAs to core competencies, independently. Participants checked the relevance of each EPA to the core competencies. Due to the familiarity of experts with the concept of competency and EPA, researchers have not defined this and allowed each of the participants to interpret the relevance of each EPA to the core competencies in their way. The degree of the relevance for each EPA with related competencies was as follows: minus for less than 50 percent, plus one for 50 to 59 percent, plus two for 60 to 69 percent, plus three for 70 to 79 percent, and plus four for more than 80 percent.

## Findings

### Step A: Reviewing the literature and conducting the interviews

In terms of presenting medical ethics programs for faculty members, 10 articles and 10 medical school websites were reviewed. Then, 60 codes were extracted using content analysis of articles and curricula.

In interviews, 14 experts were asked about the expected duties and competencies of faculty members participating in faculty development courses on medical ethics. Then, 235 codes were extracted from the interviews. Then, 295 code emerged from the data that was classified under the six categories include: teaching, and research, management of organizational ethics in healthcare, communication skills, moral reasoning, ethical policy- making and decision- making (Table 1).

**Table 1:** Summary of categories and sub-categories, as well as participants’ sample quotes about the core competencies and tasks of faculty members in medical ethics

Categories	Sub-categories	Quote Sample
Teaching	Teaching medical ethics	“Faculty members have the most significant role in teaching medical ethics to learners because learners face them daily. (No 2. Female)
	Providing educational advice in medical ethics field	“[Faculty members] should be trained in more specialized medical ethics topics that are related to their field of specialty so that they can be referred to or consulted with when needed in their group”. (No 11. Female)
	Facilitation in medical ethics	It is possible to entrust the training of undergraduate students to clinical faculty members who are not ethics experts but are familiar with ethics.” (No 5. Female)
Research	Conducting and supervising dissertations in medical ethics	“You can contact faculty members in various fields and encourage them to consider joint dissertations with students in medical ethics.” (No 4. Female)
	Conducting projects in medical ethics	“In addition to supervising research ethics committees, faculty members should consider ethical aspects in conducting and designing studies.” (No 3. Male)
	Providing advice to individuals or committees regarding codes of ethics in research	“In prescriptive or recommended, “developing codes, guidelines or instructions, outside of the target discipline is intangible and inapplicable for that target discipline’s specialists.” (No 5. Female)
	Peer reviewing projects in the field of medical ethics	“Faculty members with the competency of medical ethics can be present in research committees as peer reviewers.” (No 2. Female)
	Developing guidelines in medical ethics	“Faculty members can help by having ethical oversight in writing treatment guidelines with considering medical ethics issue.” (No 13. Female)
Management of organizational ethics in healthcare	Management of organizational ethics’ activities	“Faculty members should know the principles of management, policy-making, and policy-evaluation so that later they can ethically manage the issues happening in the public healthcare domain, such as ethical issues in quarantine, vaccination, or compulsory healthcare system programs.” (No 5. Females)
	Planning of organizational ethics’ policies	“Decision-makers should be familiar with the philosophical perspectives of ethics when decisions are made regarding the general principles of management, especially in areas such as resource allocation.”(No 12. Male)
Communication skills	Resolving conflicts based on principles of ethics	“Sometimes, patients argue with us…Someone shouts at the ethics faculty members in front of their students, and then you want them to resolve conflicts in such situations, lead and guide, and ethically solve the issues.” (No 7. Male)
	Teamwork in medical ethics field	“Teamwork skills are required to resolve ethical issues of various stakeholders.” (No 8. Male)
Moral reasoning	Critical analysis of ethical problems	... Critical thinking taught me that such actions are immoral and any moral argument about them is weak and unjustifiable.” (No 8. Male)
	Resolving ethical problems	“Faculty members should be able to review and analysis problems and challenges and make decisions to solve them.” (No 2. Female)
Ethical policy- making and decision- making	Cooperation in the dissemination of medical ethics	“If we want medical ethics and related concepts to be spread and breached in medical teams, we have no choice but to use the power and capacity of collaboration with all colleagues in the ethics field.” (No 5. Female)
	Providing advice to managers, policy-makers and planners in the field of medical ethics	“Country’s resources are limited, if we use, for example, 5% of health resources for certain patients, is it right? Or, we should spend this 5% on the common diseases of the country, to benefit more patients; Some special patients die because we did not provide facilities. The different perspectives we have are important. Managers at general levels, not at a philosophical level, but at related details (for example, justice and resource allocation) should be trained and receive a consultation, and then face problems.” (No 12. Male)
	Ethical decision-making	“Faculty members should participate hospital’s ethics committees as they can make better decisions and discuss problems. We need committee members who are scientists familiar with ethics, but we don’t have enough of such members.” (No 14. Male)
	Leadership in promoting medical ethics	“Discussing or developing medical ethics in the academic environment without the leading of stakeholders seems meaningless.” (No 12. Male)

### Step B: Holding the expert panel

At this stage, extracted tasks and core competencies were sent to the experts who attended the meeting to discuss the items. Their revisions on the quality of writing style and content for competencies were received. Moreover, based on CVR and CVI indices showed a consensus for the relevance and importance of all but one competency and discarded it from the list of competencies. Final core competencies include “Teaching medical ethics”, “Research and scholarship in the field of medical ethics”, “Communication skills”, “Moral reasoning”, “Policy-making, decision-making, and ethical leadership” (Table 2).

**Table 2:** CVIs and CVRs calculated for expected core competencies in medical ethics education

Core Competencies	CVI	CVR
Teaching of medical ethics	1	1
Research and scholarship in the field of medical ethics	1	1
Management of organizational ethics in healthcare	1	0
Communication skills	0.87	0.75
Moral reasoning	1	1
Policy-making, decision-making and ethical leadership	1	0.75

### Step C: Compile a list of EPAs

At this stage, the taskforce extracted 25 EPAs from the tasks approved in the previous phases.

### Step D: Validating the list of EPAs

The closed-ended questionnaire, which included 25 EPAs from the literature review and interview phases, was sent to 11 medical ethics professionals, to which 8 (72.2%) responded. Their completed questionnaires were analyzed, and the items with a score above 70% in terms of necessity and relevancy were retained. Analysis revealed that none of the 25 items had an importance score of less than %70, and two Items with a relevance score of less than %70 were excluded. Table 3 presents experts’ responses on the importance and relevance of expected EPAs.

**Table 3:** The percentage of the importance and relevance of expected EPAs in medical ethics

	EPAs list	Importance				Relevance			
No	Title	High	Moderate	low	No idea	High	Moderate	Low	No idea
1	Teaching medical ethics to faculty members through faculty development programs	100	0	0	0	100	0	0	0
2	Providing consultation and feedback to faculty members in the field of medical ethics	100	0	0	0	87.5	0	12.5	0
3	Direct teaching of medical ethics to students	100	0	0	0	100	0	0	0
4	Integrated teaching of medical ethics to curricula through role modeling, etc.	87.5	12.5	0	0	87.5	12.5	0	0
5	Doing Facilitation in various situations of medical ethics education	75	12.5	0	12.5	75	12.5	0	12.5
6	Identifying and determining research priorities in medical ethics	87.5	12.5	0	0	75	37.5	0	0
7	Designing, implementation and Collaboration in medical ethics projects	100	0	0	0	100	0	0	0
8	Publishing and disseminating findings of medical ethics projects	87.5	12.5	0	0	87.5	12.5	0	0
9	Providing consultation to research ethics committees	100	0	0	0	100	0	0	0
10	Ethical guidance and supervision of research projects	87.5	12.5	0	0	87.5	12.5	0	0
11	Ethical peer reviewing and critique of research projects	87.5	12.5	0	0	100	0	0	0
12	Developing codes of conduct and ethical guidelines in research, treatment, etc.	87.5	12.5	0	0	100	0	0	0
13	Managing and directing planning activities in medical ethics field	87.5	12.5	0	0	62.5	37.5	0	0
14	Providing ethical guidance and supervision of guidelines	70	12.5	12.5	0	75	12.5	12.5	0
15	Leading change management in promoting medical ethics at micro and macro levels	87.5	12.5	0	0	75	12.5	12.5	0
16	Decision-making on equitable allocation of resources	87.5	0	12.5	0	62.5	37.5	0	0
17	Managing the conflict in communications and interactions based on ethical principles	87.5	0	12.5	0	87.5	0	12.5	0
18	Collaborating in team activities related to the field of medical ethics	100	0	0	0	100	0	0	0
19	Identifying and critically analyzing ethical issues and dilemma	100	0	0	0	100	0	0	0
20	Providing evidence-based solutions to ethical issues and challenges	100	0	0	0	100	0	0	0
21	Conducting ethical reflection on personal and organizational activities	100	0	0	0	100	0	0	0
22	Planning, collaboration and directing activities related to the development and promotion of medical ethics in healthcare system	87.5	12.5	0	0	100	0	0	0
23	Providing advice to managers, policy makers and planners in promoting medical ethics programs	75	12.5	0	12.5	87.5	0	0	12.5
24	Guiding organizational ethics committees	87.5	12.5	0	0	100	0	0	0
25	Evaluating the quality of ethical decisions and policies	87.5	12.5	0	0	100	0	0	0

### Step E: Mapping EPAs to core competencies

Table 4 presents the relationship between EPAs and core competencies of medical ethics for faculty members.

**Table 4:** Mapping of EPAs to core competencies in medical ethics training in faculty development programs

No.	EPAs Titles	Teaching	Research and scholarship	Communication skills	Moral reasoning	Policy-making, decision-making, and leadership
1	Teaching medical ethics to faculty members through faculty development programs	++++	-	+++	+++	-
2	Providing consultation and feedback to faculty members in the filed of medical ethics	-	-	++	++++	-
3	Direct teaching of medical ethics to students	++++	-	+	+	-
4	Integrated teaching of medical ethics to curricula through role modeling, etc.	+++	-	++++	+++	-
5	Doing Facilitation in various situations of medical ethics education	++	-	++++	+	-
6	Identifying and determining research priorities in medical ethics	-	++++	+	-	++
7	Designing, implementation and collaboration in medical ethics projects	-	++++	+	-	+
8	Publishing and disseminating findings of medical ethics projects	-	+++	+	-	+++
9	Providing consultation to research ethics committees	-	-	+++	++++	+
10	Ethical guidance and supervision of research projects	-	+	++	+++	-
11	Ethical peer reviewing and critique of research projects	-	-	+	+++	++++
12	Developing codes of conduct and ethical guidelines in research, treatment, etc.	-	++++	-	+	++++
13	Providing ethical guidance and supervision of guidelines	-	+	-	+	++
14	Leading change management in promoting medical ethics at micro and macro levels	-	-	++	++	++++
15	Managing the conflict in communications and interactions based on ethical principles	-	-	++++	++++	++
16	Collaborating in team activities related to the field of medical ethics	-	-	++++	+	+
17	Identifying and critically analyzing ethical issues and dilemma	-	++	-	++++	+
18	Providing evidence-based solutions to ethical issues and challenges	-	+	-	++++	+
19	Conducting ethical reflection on personal and organizational activities	-	-	-	++++	+
20	Planning, collaboration and directing activities related to the development and promotion of medical ethics in healthcare system	-	-	+++	+	++++
21	Providing advice to managers, policy makers and planners in promoting medical ethics programs	-	-	++++	++++	++++
22	Guiding organizational ethics committees	-	-	++	++++	++
23	Evaluating the quality of ethical decisions and policies	-	-	-	+++	++++

## Discussion

Empowerment of human resources is considered one of the main strategies to achieve resources with appropriate competencies, to lead the organizational change process [26]. Faculty development programs intend to enhance professional knowledge and skills to adapt to the growing changes; training and development of faculty members play a valuable role in balancing professional knowledge and skills [27]. Research showed that a lack of medical ethics knowledge and awareness is a significant challenge leading to unethical practices [28, 29].

In the present study, teaching skills were identified as a core competency for faculty members in medical ethics education. Creating educational opportunities is detrimental to empowering faculty members who can teach medical ethics in active ways and relevant to their field’s topics. Furthermore, for integrated medical ethics teaching, faculty members should teach professional behaviours to students in various educational opportunities through courses as well as act as role models [30]. The role of medical teachers as creditable role models for students is necessary for teaching professional and ethical behaviours [11, 31]. In the study by Bligh *et al.*, the following core competencies were considered required for medical teachers: teaching skills, effective communication skills, professionalism, expert knowledge, and role modeling [32].

Research and scholarship in the field of medical ethics are considered as core competencies for faculty members in this study. In our study, faculty members must be competent to design and conduct high-quality original research, developmental and prescriptive research. In this category, the set of outcomes mainly related to determining research priorities, methodology design, implementation, application and dissemination of results as well as evidence-based practice in the field of medical ethics is required. Scholarship highlighted encouraging the participation of faculty members in developing their innovative practice in the medical ethics. Thomas (2003) recommended that providing professional incentives that value scholarship in medical ethics, even when it is not a faculty member’s primary research area, is necessary for promoting scholarship activities in medical ethics [33]. In this study, besides the research skills to generate the medical ethics studies, the scholarship capability to explore interventions for solving ethical dilemmas and challenges, as well as the ability of informed decision making to choose the best professional practice in workplace settings were emphasized.

Effective communication skills with different stakeholders to implement and teach medical ethics at the interpersonal and team level were identified as another core competency in the present study. According to our findings, to convey ethical values ​​and beliefs to medical students in all three areas of knowledge, attitude and behavior, and institutionalize medical ethics in the healthcare system, medical teachers should have high-level effective communication skills [34].

According to the current study, Moral reasoning and Policy-making, decision making and ethical leadership are other core competencies for medical ethics faculty members, which other studies referred to it as a multidimensional skill consisting of individual and organizational competencies [35]. Despite the importance of acquiring moral reasoning competence, the evidence showed the lack of moral reasoning approaches in graduated physicians in dealing with patients [36]. Moral reasoning capability is required for development of professionalism in various dimensions [37]. In this regard, conducting educational interventions like providing faculty development programs to enhance role modeling and feedback by faculty members that positively influence students’ moral development can be suggested [38]. Policy-making, decision-making, and ethical leadership competencies have not only been used for resolving ethical conflicts created in the educational system along with communication skills but also to guide policies and procedures to evaluate educational opportunities that incorporate such abilities in educational programs are necessary [39]. Furthermore, based on our findings, the competency of managing organizational ethics in healthcare did not earn the necessary votes to be considered as a core competency because participants interpreted it as the capability of management at different levels in medical schools.

To implement competencies in practice and workplace, EPAs in the competency-based curriculum should be used. EPAs translate essential competencies for medical education related to required tasks and duties [40]. Faculty members’ opinions on EPAs showed that most of the tasks, except two items, assigned to faculty members after attending medical ethics faculty development programs, received essential scores in terms of necessity and relevance to the core competencies. These two items were as follows: “Decision-making on resource allocation”, and “Managing and directing planning activities in the medical ethics field." These two items, possibly, due to the direct role of managers and educational administrators in promoting it at the organizational level, are less significant in faculty members’ professional duties. According to the findings, the score of moral reasoning competency was equal to or greater than 70% of those of other 11 EPAs, indicating the importance of this competency and the need to address it further; meanwhile, in existing studies, this competency and tools for its measurement were discussed [40, 41].

Studies on EPAs required for faculty members in the field of medical ethics were not adequate, whereas such studies were required to conduct practical and edifying courses for faculty development programs. Moreover, the participation of various experts from the clinical and medical ethics departments and educational administrators in this study, especially those who had research or activities in the field of medical ethics, was valuable to obtain various stakeholders’ viewpoints. In addition, a multi-stage method together with quantitative and qualitative study methods helped explain the study topic. As a limitation, few articles addressed medical ethics curricula or faculty members’ tasks in medical ethics. Another limitation is the need to change the perspective on the concept of EPA qualification for teachers before starting their teaching, which was difficult to define during the study by the participants.

## Conclusion

Medical teachers are significantly effective in moralizing the healthcare system and faculty members should attain core competencies and EPAs for medical ethics education. In this study, the core competencies components of faculty members in the field of medical ethics in five core competencies domains have been defined as teaching skills, research and scholarship, communication skills, moral reasoning, and decision-making, policy-making and ethical leadership. Faculty development programs can be designed in the field of medical ethics for faculty members to help them acquire core competencies and EPAs.

## Data Availability

The datasets used and/or analysed during the current study available from the corresponding author on reasonable request.

## Abbreviations

EPA: Entrustable Professional Activity
CVI: Content Validity Index
CVR: Content Validity Ratio
